# Association of elevated serum aminotransferase levels with chronic kidney disease measures: hispanic community health study/study of latinos

**DOI:** 10.1186/s12882-021-02483-y

**Published:** 2021-09-07

**Authors:** Celestin Missikpode, Holly Kramer, Scott J. Cotler, Ramon Durazo-Arvizu, James P. Lash, Eric Kallwitz, Jianwen Cai, Mark H. Kuniholm, Sylvia E. Rosas, Ana C. Ricardo, Gregory A. Talavera, Leopoldo Raij, Amber Pirzada, Martha L. Daviglus

**Affiliations:** 1 Institute for Minority Health Research, University of Illinois at Chicago, 1819 W Polk St, Ste 246, IL 60612 Chicago, USA; 2 Department of Medicine, Division of Nephrology and Hypertension, Loyola University Chicago Health Sciences Campus, IL Maywood, USA; 3 Department of Public Health Sciences, Loyola University Chicago Health Sciences Campus, IL Maywood, USA; 4 Department of Medicine, Division of Hepatology, Loyola University Chicago Health Sciences Campus, Illinois Maywood, USA; 5 Department of Medicine, University of Illinois at Chicago, Illinois Chicago, USA; 6 Collaborative Studies Coordinating Center, Department of Biostatistics, University of North Carolina at Chapel Hill, North Carolina Chapel Hill, USA; 7 Department of Epidemiology and Biostatistics, University at Albany, NY Rensselaer, USA; 8 Kidney and Hypertension Unit, Joslin Diabetes Center, Harvard Medical School, MA Boston, USA; 9 South Bay Latino Research Center, Graduate School of Public Health, San Diego State University, San Diego, USA; 10 Department of Medicine, Division of Nephrology and Hypertension, University of Miami Miller School of Medicine, Miami, USA

**Keywords:** NAFLD, Chronic kidney disease, Obesity, Race/ethnicity, Aminotransferase levels, Hispanics/Latinos

## Abstract

**Background:**

Previous studies have shown an association between non-alcoholic fatty liver disease (NAFLD) and chronic kidney disease (CKD), but it is unclear whether the association is independent of metabolic syndrome.

**Methods:**

Data from 13,006 participants aged 18 to 74 years in the Hispanic Community Health Study/Study of Latinos (HCHS/SOL) without viral hepatitis, excessive alcohol consumption, or high transferrin saturation levels were analyzed. Suspected NAFLD was defined as presence of sex-specific elevations in serum aminotransferase levels (aspartate aminotransferase (AST) > 37 U/L or alanine aminotransferase (ALT) > 40 U/L for men and AST or ALT > 31 U/L for women). Logistic regression was used to examine cross-sectional associations of elevated serum aminotransferase levels with low estimated glomerular filtration rate (eGFR < 60 ml/min/1.73 m^2^ based on cystatin C), and with high urinary albumin-to-creatinine ratio (UACR) (> 17 mg/g in men and > 25 mg/ g in women) in separate models adjusting for demographic characteristics and metabolic syndrome.

**Results:**

Mean (SD) age was 41 (0.27) years, and 45 % were male. Elevated serum aminotransferase levels were noted in 18.8 % of the population and were associated with greater odds of high UACR (OR = 1.31; 95 % CI = 1.10, 1.56) after adjusting for demographic characteristics; this association became non-significant after adjustment for metabolic syndrome (OR = 1.11, 95 % CI = 0.92, 1.33). In contrast, elevated serum aminotransferase levels were not associated with low eGFR (odds ratio (OR) = 0.73; 95 % confidence interval (CI) = 0.45, 1.18) after adjusting for covariates.

**Conclusions:**

In this sample of diverse U.S. Hispanic Latino adults, elevated serum aminotransferase levels were not independently associated with measures of CKD.

**Supplementary Information:**

The online version contains supplementary material available at 10.1186/s12882-021-02483-y.

## Background

Non-alcoholic fatty liver disease (NAFLD), a heterogeneous disorder ranging from non- alcoholic steatosis to non-alcoholic steatohepatitis, is rapidly becoming a leading cause of chronic liver disease [1]. Data from National Health and Nutrition Examination (NHANES) surveys showed that the contribution of NAFLD towards the burden of chronic liver disease increased from 46.8 % in 1988–1994 to 75.1 % in 2005–2008 [1]. During this period, the prevalence of metabolic syndrome (e.g., obesity and diabetes) also increased [1].

Prior studies have reported that Hispanics/Latinos have a high burden of NAFLD as compared to other racial/ethnic groups [2, 3]. A meta-analysis of population-based cohorts found that nearly 1 in 4 Hispanics/Latinos have NAFLD, and the risk of NAFLD is 36 % higher among Hispanics than non-Hispanic Whites [3]. In parallel to the high NAFLD burden, Hispanics/Latinos also experience a substantial burden of chronic kidney disease (CKD) [4–6]. The population-based Hispanic Community Health Study/Study of Latinos (HCHS/SOL) reported that prevalence of CKD among Hispanics/Latinos was 13.7 % [6].

Evidence suggests an association between NAFLD and CKD [7–10]; however, data for the association independent of metabolic syndrome are not consistent. While some studies have reported an association between NAFLD and CKD independent of metabolic syndrome [7–9], others have not observed such an association [9, 10]. Understanding whether NAFLD and CKD share metabolic syndrome as a common pathway is important as this can have major implications. For example, interventions can be geared toward metabolic syndrome to reduce the burden of both NAFLD and CKD if the association between these disorders is explained by metabolic syndrome.

We used data from the HCHS/SOL to examine the association between NAFLD (defined as elevated serum aminotransferase levels) and prevalence of CKD. As an exploratory analysis, we also tested whether this association differs by sex and Hispanic/Latino background given that elevated serum aminotransferase levels are sex-specific, and that the Hispanic/Latino population is a heterogenous group with a non-uniform distribution of NAFLD and CKD.

## Methods

### Study population

The details of the design and implementation of HCHS/SOL cohort have been previously published [11, 12]. Briefly, HCHS/SOL is a community-based cohort study of 16, 415 self-identified Hispanic/Latino adults aged 18–74 years from randomly selected households in four U.S. field centers (Chicago, Bronx, Miami and San Diego) who underwent a comprehensive baseline examination in 2008 to 2011. The HCHS/SOL cohort includes participants of Mexican, Puerto-Rican, Cuban, Central American, Dominican, South American, and other/mixed backgrounds. The study was approved by the Institutional Review Board at each participating institution, and all participants provided written informed consent.

**Fig. 1:**
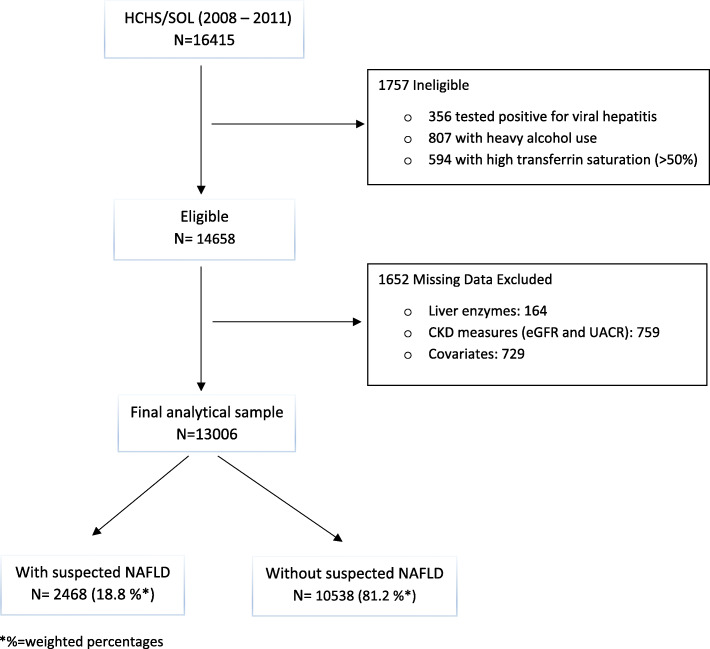
Flowchart of analytical sample selection

For the present analyses, of the 16,415 HCHS/SOL participants, we excluded 356 individuals seropositive for hepatitis C virus (anti-HCV) or hepatitis B virus (HBsAg), 807 individuals with excessive alcohol use (alcohol consumption ≥ 7 drinks per week in women, or ≥ 14 drinks per week in men). A questionnaire was administered to assess alcohol consumption. Current alcohol drinkers were asked to provide the amount and frequency of consumption of alcoholic beverages including red wine, white wine, beer, liquor, spirits, or mixed drinks in a week. Participants were classified into three groups based on gender-specific cutoffs for weekly alcohol consumption established by the National Institute for Alcohol Abuse and Alcoholism to assess the risk of alcohol use disorder: no risk = never used alcohol, low risk = current use < 7 drinks/week (women), < 14 drinks /week (men), and at-risk = ≥ 7 drinks/week (women), ≥ 14 drinks /week (men) [13]. We also excluded 594 with transferrin saturation > 50 % as evidence of iron overload as described previously [14]. We further excluded individuals with missing information on liver enzymes (n = 164), CKD measures (n = 759), and covariates (n = 729). This resulted in an analytical sample of 13,006 individuals (Fig. 1).

### Study variables

#### Elevated Serum Aminotransferase Levels

Blood samples were collected following standardized procedures after an eight-hour fast and were centrifuged and frozen within 45 min of collection. Radiologic liver exams were not available in HCHS/SOL. Consistent with a previous report from HCHS/SOL, we defined suspected NAFLD using serum aspartate aminotransferase (AST) and alanine aminotransferase (ALT) [14]. Thus, NAFLD was suspected if AST was greater than 37 U/L or ALT greater than 40 U/L in men, and if AST or ALT was greater than 31 U/L in women [14]. In sensitivity analyses, we also used arbitrary higher ALT thresholds to define elevated ALT levels as follows: mild if ALT > 50 U/L in men and ALT > 40 U/L in women; moderate if ALT > 60 U/L in men and ALT > 50 U/L in women; severe if ALT > 70 U/L in men and ALT > 60 U/L in women.

#### Fatty liver index and liver fibrosis score

We also identified NAFLD by calculating Fatty Liver Index (FLI) as follows: Fatty Liver Index (FLI)= ${e}^{y}$/ (1 + ${e}^{y}$) × 100; where y = 0.953 × ln(triglycerides, mg/dL) + 0.139 × BMI, kg/m2 + 0.718 × ln (GGT, U/L) + 0.053 × waist circumference, cm – 15.745) [15]. A FLI value of 60 or greater is considered indicative of steatosis [15]. We further predicted advanced fibrosis by calculating liver fibrosis score 4 (FIB-4) as follows: FIB4 = [Age × AST] / [Platelets × √ALT]) [16]. Consistent with prior studies, we classified participants into low (FIB-4 < 1.3), moderate (FIB-4: 1.3–2.67), and high (FIB-4 > 2.67) fibrosis score groups [17]. Fibrosis scores greater than 2.67 generally indicate advanced liver fibrosis [17].

#### Chronic Kidney Disease Measures

CKD measures included low estimated glomerular filtration rate (eGFR < 60 ml/min/1.73 m^2^), and the presence of albuminuria defined as increased urine albumin-to-creatinine ratio (UACR) measured in a spot urine specimen. For our main analysis, eGFR was calculated using a formula based on cystatin C because creatinine-based GFR estimating equations do not perform well in the presence of liver disease [18]. In sensitivity analyses, we also calculated eGFR using the CKD-EPI creatinine-cystatin C equation [18]. HCHS/SOL measured serum creatinine on a Roche Modular P Chemistry Analyzer using a creatinase enzymatic method (Roche Diagnostics, Indianapolis, IN 46,250), and serum cystatin C was measured using a turbidimetric method on the Roche Modular P Chemistry Analyzer (Gentian AS, Moss, Norway). To account for differences in creatinine excretion between men and women, sex-specific cut-points for increased UACR were used, i.e., > 17 mg/g in men and > 25 mg/ g in women [19, 20].

### Covariates

Participants completed standardized interviews to ascertain demographic data, health behaviors, and venous blood and urine specimen collection. Physical exams included height, weight, waist circumference, and 3 seated brachial blood pressure measurements obtained after a 5-minute rest period. Hypertension was defined as systolic blood pressure ≥ 140 mm Hg, diastolic blood pressure ≥ 90 mm Hg, or use of antihypertensive medications [21]. Diabetes was defined as fasting glucose ≥ 126 mg/dL, random glucose ≥ 200 mg/dL, post-oral glucose tolerance test (OGTT) ≥ 200 mg/dL, hemoglobin A1C ≥ 6.5 %, or use of insulin or other anti-diabetic medication [22]. Low high-density lipoprotein (HDL) cholesterol was defined as HDL cholesterol < 40 mg/dL in men and < 50 mg/dL in women [23]. High triglyceride level was defined as serum triglycerides ≥ 150 mg/dL [24]. The homeostasis model assessment of insulin resistance (HOMA-IR) was calculated as the product of fasting glucose (mg/dL) and fasting insulin (mU/L) divided by 405 (HOMA-IR = fasting glucose (mg/dL) X fasting insulin (mU/L)/405) [25]. Additional covariates considered in this study were age, sex, self-reported Hispanic/Latino background, educational attainment (less than high school, high school or GED, more than high school), health insurance coverage, cigarette smoking status (never, former, current). Data were also collected on medications including angiotensin converting enzyme inhibitors /angiotensin receptor blockers (ACEI/ARB) and corticosteroids. The HCHS/SOL asked participants to bring in all prescribed or over-the-counter medications and drug products were assigned to their medication classes based on their generic ingredients. The use of steatogenic medications such as tamoxifen, amiodarone, and valproic acid in our study population was very low (< 0.01 %). Among U.S. Hispanic/Latinos, the three predominant sources of ancestry correspond to European, Native American and African founder populations, and ancestry/background was grouped into two major arms representing Mainland (Mexican, Central or South American) and Caribbean heritage (Puerto Rican, Dominican or Cuban) [26].

### Statistical Analysis

The analysis accounted for the complex survey design and adjusted for sampling probability and nonresponse. Survey-specific procedures were used to account for the 2-stage sampling design, stratification, and clustering [11, 12]. Baseline characteristics and prevalence of CKD measures were examined by presence of elevated aminotransferase levels. Continuous variables were compared using linear regression (proc surveyreg) and categorical variables were compared using the Rao-Scott chi-squared test.

Survey-weighted logistic regression models were used to investigate associations of elevated serum aminotransferase levels with each of the two CKD measures separately. We adjusted for covariates in a progressive fashion: (Model 1) age, sex, Hispanic/Latino background, and study site; (Model 2) additional adjustment for metabolic syndrome; (Model 3) further adjusted for education attainment, cigarette smoking, alcohol consumption, having health insurance, and use of corticosteroids and angiotensin converting enzyme inhibitors/angiotensin receptor blockers). An interaction between elevated serum aminotransferase levels and sex and Hispanic/Latino background, respectively, was explored in the final model. In sensitivity analyses, we re-analyzed the data using serum aminotransferase as continuous variables. All statistical analyses were conducted using SAS version 9.4 (SAS Institute, Cary, NC).

## Results

### Characteristics of study population

All estimates are weighted for the complex HCHS/SOL survey design and sampling. The study population had a mean (SD) age of 41 (0.27) years, and 45 % were male. Elevated serum aminotransferase levels were present in 18.8 % of the study population, 22.8 % in men and 15.5 % in women. The prevalence of low eGFR was 2.7 % and that of increased UACR was 12.4 % in men and women. Characteristics of the study population by presence/absence of elevated aminotransferase levels are presented in Table 1. Individuals with elevated serum aminotransferase levels were more likely to be male, of Mexican background, obese, and less likely to have health insurance. In addition, they were more likely to have metabolic syndrome, diabetes, hypertension, low HDL cholesterol, and high triglycerides. This group also had significantly higher waist circumference and HOMA-IR scores than those without elevated aminotransferase levels. Individuals with and without elevated aminotransferase levels had similar distribution of age, education attainment, cigarette smoking status, and ACEI/ARB use.

**Table 1:** Characteristics of HCHS/SOL Population (2008–2011) by Presence of Elevated Serum Aminotransferase Levels (*n* = 13,006)

Variables	Elevated serum Aminotransferase Levels (*n* = 2,468) % or mean (SD)	No elevated Serum Aminotransferase Levels (*n* = 10,538) % or mean (SD)	*P*-value
*N* = 13,006	2468	10,538	
Age, years	40.8 (0.45)	41.5 (0.29)	0.098
Male, %	54.7	42.8	< 0.0001
Hispanic/Latino background, %			
Dominican	7.1	10.3	0.0002
Central American	8.7	7.4	0.057
Cuban	18.6	21.2	0.039
Mexican	43.8	36.4	< 0.0001
Puerto Rican	13.1	15.7	0.048
South American	5.1	5.2	0.910
More than one/Other heritage	3.6	3.8	0.754
Less than high school diploma, %	31.7	32.2	0.697
Having health Insurance, %	43.7	52.4	< 0.0001
Current smoker, %	17.8	18.8	0.136
Body mass index, kg/m2	31.7 (0.17)	29.0 (0.10)	< 0.0001
Obesity (BMI ≥ 30 kg/m2), %	56.2	37.1	< 0.0001
ACE inhibitor or ARB use, %	8.5	8.9	0.674
Metabolic syndrome	49.0	30.1	< 0.0001
Metabolic syndrome components			
Waist circumference, cm	102.9 (0.43)	96.4 (0.24)	< 0.0001
HOMA-IR	5.2 (0.12)	3.1 (0.05)	< 0.0001
Diabetes, %	20.1	14.3	< 0.0001
Hypertension, %	24.2	21.9	0.068
Low HDL cholesterol, %	53.3	40.0	< 0.0001
High triglyceride level, %	44.7	25.8	< 0.0001
eGFR (mL/min/1.73 m2)	97.5 (0.60)	95.2 (0.38)	0.002
eGFR < 60 ml/min, %	1.7	3.0	0.013
UACR (mg/g)	28.1 (3.8)	31.3 (2.6)	0.497
Increased UACR, %	14.6	11.9	0.007

All data shown as weighted % or mean (SD)

Elevated aminotransferase levels defined as aspartate aminotransferase (AST) > 37 U/L or alanine aminotransferase (ALT) > 40 U/L in men and AST or ALT > 31 U/L in women

*ACEI/ARB* angiotensin-converting enzyme inhibitor/angiotensin receptor blocker; *HOMA-IR* homeostasis model assessment of insulin resistance; *eGFR* estimated glomerular filtration rate (eGFR calculated from CKD-EPI cystatin C equation); *UACR* urine albumin-to-creatinine ratio; Increased UACR defined as UACR > 17 mg/g in men and > 25 mg/ g in women

### Association of elevated aminotransferase levels with low eGFR and increased UACR

Compared to individuals without elevated aminotransferase levels, those with elevated aminotransferase levels had significantly lower prevalence of low eGFR (1.7 % vs. 3.0 %, *p* = 0.013) in unadjusted analysis. Table 2 shows association between elevated aminotransferase levels and prevalence of low eGFR. In unadjusted survey logistic regression, elevated aminotransferase levels were significantly associated with lower odds of low eGFR (OR = 0.55; 95 % CI = 0.34, 0.88, *p* = 0.014). The association became nonsignificant after adjusting for demographic characteristics including age, sex, Hispanic/Latino background, and study site (OR = 0.73, 95 %CI = 0.45, 1.19). We found no gender differences in elevated aminotransferase levels’ relationship to low eGFR (p-value for interaction = 0.69), and no evidence of an interaction between elevated aminotransferase levels and Hispanic/Latino background (*p* = 0.53).

**Table 2:** Odds Ratio and 95 % confidence intervals of the association of elevated aminotransferase levels and fatty liver index (FLI) with low eGFR (eGFR calculated from CKD-EPI cystatin C equation) and increased urine albumin-to-creatinine ratio (UACR)

Regression models	Low eGFR		increased UACR	
	OR and 95 %CI	*P*-value	OR and 95 %CI	*P*-value
Elevated aminotransferase levels				
Unadjusted model	0.55 (0.34, 0.88)	0.014	1.27 (1.07, 1.51)	0.007
Model 1	0.73 (0.45, 1.19)	0.208	1.31 (1.10, 1.56)	0.003
Model 2	0.77 (0.47, 1.25)	0.287	1.11 (0.92, 1.33)	0.272
Model 3	0.73 (0.45, 1.18)	0.201	1.13 (0.94, 1.35)	0.189
Elevated FLI				
Unadjusted model	1.08 (0.80, 1.46)	0.607	1.85 (1.58, 2.16)	< 0.0001
Model 1	0.76 (0.56, 1.02)	0.069	1.60 (1.37, 1.87)	< 0.0001
Model 2	0.82 (0.60, 1.13)	0.231	1.13 (0.96, 1.33)	0.1348
Model 3	0.77 (0.56, 1.05)	0.094	1.13 (0.96, 1.33)	0.1462

Elevated aminotransferase levels defined as aspartate aminotransferase (AST) > 37 U/L or alanine aminotransferase (ALT) > 40 U/L in men and AST or ALT > 31 U/L in women

Elevated Fatty Liver Index (FLI) defined as FLI ≥ 60

Low eGFR defined as eGFR < 60 ml/min/1.73 m^2^

Increased UACR defined as UACR > 17 mg/g in men and > 25 mg/ g in women

Model 1 adjusted for age, sex, Hispanic/Latino background, and study site

Model 2 adjusted for age, sex, Hispanic/Latino background, study site, and metabolic syndrome

Model 3 adjusted for age, sex, Hispanic/Latino background, study site, metabolic syndrome, education attainment, alcohol consumption, cigarette smoking, having health insurance, use of angiotensin converting enzyme inhibitors/angiotensin receptor blockers, and corticosteroids

Low eGFR outcome: interaction sex*aminotransferase *p* = 0.69 (Model 3); interaction background*aminotransferase *p* = 0.53 (Model 3)

Increased UACR outcome: interaction sex*aminotransferase: *p* = 0.75 (Model 3); interaction background*aminotransferase: *p* = 0.73 (Model 3)

In contrast to low eGFR, individuals with elevated aminotransferase levels had significantly higher prevalence of increased UACR than those without elevated aminotransferase levels (14.6 % vs.11.9 %, *p* = 0.007). The unadjusted odds ratio for the presence of increased UACR among individuals with (vs. without) elevated aminotransferase levels was 1.27 (95 % CI: 1.07–1.51, *p* = 0.007) (Table 2). After adjusting for age, sex, Hispanic/Latino background, and study site, individuals with elevated aminotransferase levels remained at increased odds of increased UACR (OR = 1.31, 95 % CI: 1.10–1.56, *p* = 0.003). However, the association was attenuated after further adjustment for metabolic syndrome (OR = 1.11, 95 % CI: 0.92–1.33, *p* = 0.27). Of note, this reduction in the magnitude of the association was mostly explained by hypertension, diabetes, and insulin resistance (Table 3). We noted no gender differences in elevated aminotransferase levels’ relationship to increased UACR (*p*-value for interaction = 0.75). Similarly, there was no evidence of interaction between elevated aminotransferase levels and Hispanic/Latino background (*p* = 0.73).

**Table 3:** Odds Ratio and 95 % confidence intervals of the association between metabolic syndrome and chronic kidney disease measures

	OR and 95 %CI	
	eGFR < 60 ml/min/1.73 m^2^	UACR > 17 mg/g in men and > 25 mg/ g in women
Metabolic syndrome		
No	1.00	1.00
Yes	0.77 (0.55, 1.06)	2.20 (1.88, 2.59)
Metabolic syndrome components		
Diabetes	0.99 (0.67, 1.44)	2.53 (2.10, 3.04)
Hypertension	1.13 (0.78, 1.62)	3.04 (2.44, 3.78)
HOMA-IR	1.02 (1.00, 1.03)	1.04 (1.02, 1.06)
Low HDL cholesterol	0.93 (0.67, 1.30)	1.17 (0.99, 1.37)
High triglyceride	0.73 (0.52, 1.04)	1.12 (0.95, 1.32)
Waist circumference	0.98 (0.97, 0.99)	1.00 (0.99, 1.01)

Model adjusted for age, sex, Hispanic/Latino background, study site, education attainment, alcohol consumption, cigarette smoking, having health insurance, use of angiotensin converting enzyme inhibitors/angiotensin receptor blockers, and corticosteroids. In addition, metabolic syndrome components adjusted for each other

### Sensitivity analyses

**Fig. 2:**
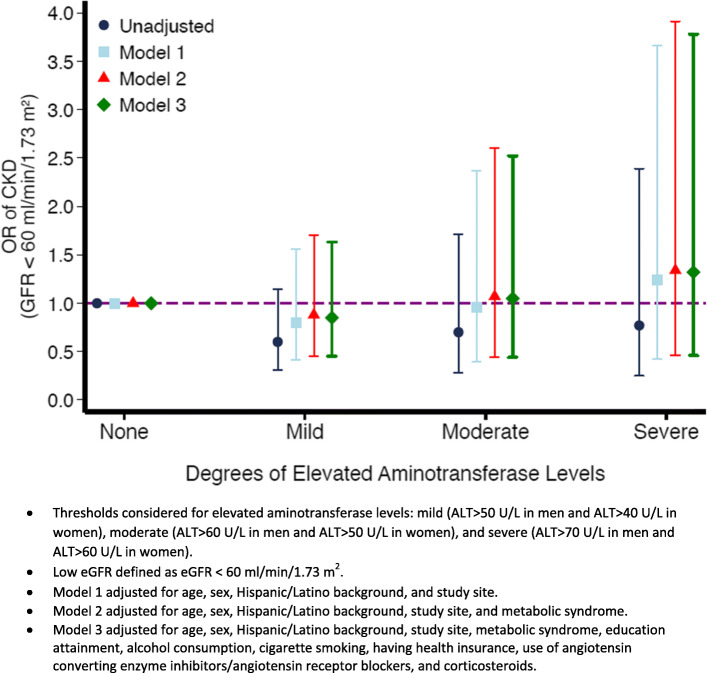
Odds Ratio and 95 % confidence intervals of the association between different degrees of elevated aminotransferase levels and low eGFR (eGFR calculated from CKD-EPI cystatin C equation)

**Fig. 3:**
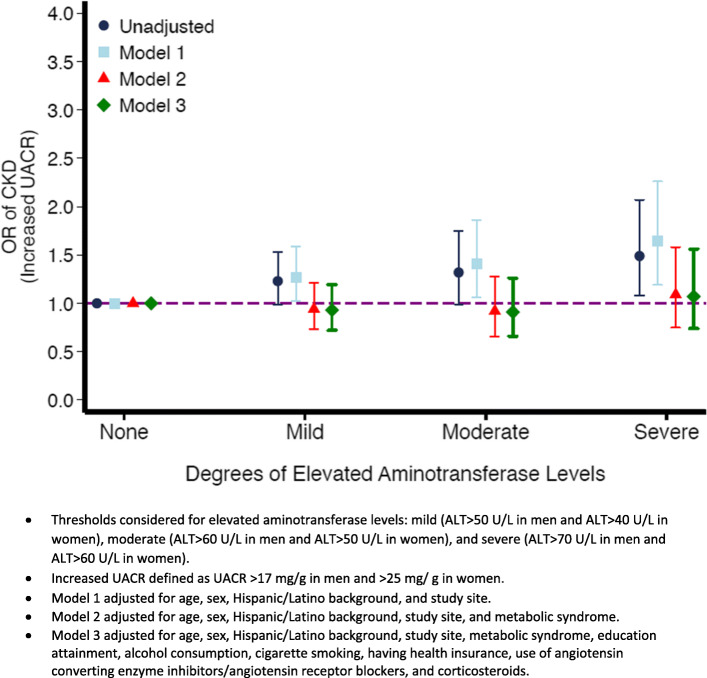
Odds Ratio and 95% confidence intervals of the association between different degrees of elevated aminotransferase levels and increased UACR

Association between degree of elevated ALT levels and CKD measures. Figure 2 shows the association between different degrees of elevated ALT levels and low eGFR. Regardless of the ALT threshold used, elevated ALT levels were not associated with low eGFR both in the unadjusted and adjusted models. However, with moderate and severe ALT thresholds (Fig. 3), elevated ALT levels were associated with higher odds of increased UACR after adjusting for demographic characteristics (OR = 1.40, 95 % CI: 1.06–1.86 and OR = 1.63, 95 % CI: 1.18–2.25, respectively). However, accounting for metabolic syndrome again attenuated these associations (OR = 1.12, 95 % CI: 0.83–1.51 and OR = 1.30, 95 % CI: 0.92–1.82 for moderate and severe ALT thresholds, respectively).

We further examined the association between elevated ALT levels and low eGFR, where eGFR was calculated from the CKD-EPI creatinine-cystatin C equations, and the results were similar (Supplemental Table 1).

We also investigated the associations between serum aminotransferase and CKD measures, where aminotransferase levels were analyzed as continuous variables. The patterns of the associations remained identical to those reported above (Supplemental Table 2).

### Association of fatty liver index and liver fibrosis score with low eGFR and increased UACR

The prevalence of elevated fatty liver index (FLI ≥ 60) was 47.2 % (SD = 0.72). The association between fatty liver index and CKD measures corroborates the results obtained from the analysis of aminotransferase levels and CKD measures (Table 2). Specifically, elevated FLI was associated with increased UACR after adjusting for demographic factors, however, the association became nonsignificant after adjusting for metabolic syndrome. There was no association between elevated FLI and low eGFR (Table 2).

Table 4 shows the association of liver fibrosis score (FIB-4) with low eGFR and increased UACR. The prevalence of moderate FIB-4 score and high FIB-score was 12.5 and 1 %, respectively. Only advanced liver fibrosis (high FIB-4) was significantly associated with increased UACR (OR 2.54, 95 % CI 1.56–4.13) after adjustment for metabolic syndrome and other risk factors (Model 3). No significant association was found with low eGFR.

**Table 4:** Odds Ratio and 95 % confidence intervals of the association between liver fibrosis score (FIB-4) and low eGFR (eGFR calculated from CKD-EPI cystatin C equation) and increased urine albumin-to-creatinine ratio (UACR)

Regression models	Weighted % (SD)	Low eGFR	Increased UACR
		OR and 95 %CI	OR and 95 %CI
Unadjusted model			
Low FIB-4	86.5 (0.45)	1.00	1.00
Moderate FIB-4	12.5 (0.43)	7.90 (5.73, 10.89)	1.87 (1.58, 2.23)
High FIB-4	1.0 (0.13)	5.38 (1.99, 14.53)	4.46 (2.82, 7.05)
Model 1			
Low FIB-4	86.5 (0.45)	1.00	1.00
Moderate FIB-4	12.5 (0.43)	1.37 (0.98, 1.93)	1.12 (0.92, 1.36)
High FIB-4	1.0 (0.13)	0.67 (0.24, 1.90)	2.47 (1.54, 3.96)
Model 2			
Low FIB-4	86.5 (0.45)	1.00	1.00
Moderate FIB-4	12.5 (0.43)	1.35 (0.96, 1.90)	1.18 (0.97, 1.43)
High FIB-4	1.0 (0.13)	0.65 (0.23, 1.83)	2.62 (1.63, 4.20)
Model 3			
Low FIB-4	86.5 (0.45)	1.00	1.00
Moderate FIB-4	12.5 (0.43)	1.31 (0.93, 1.83)	1.15 (0.94, 1.41)
High FIB-4	1.0 (0.13)	0.60 (0.21, 1.75)	2.54 (1.56, 4.13)

Liver fibrosis score (FIB-4): low (FIB-4 < 1.3), moderate (FIB-4: 1.3–2.67), and high (FIB-4 > 2.67)

Low eGFR defined as eGFR < 60 ml/min/1.73 m^2^

Increased UACR defined as UACR > 17 mg/g in men and > 25 mg/ g in women

Model 1 adjusted for age, sex, Hispanic/Latino background, and study site

Model 2 adjusted for age, sex, Hispanic/Latino background, study site, and metabolic syndrome

Model 3 adjusted for age, sex, Hispanic/Latino background, study site, metabolic syndrome, education attainment, alcohol consumption, cigarette smoking, having health insurance, use of angiotensin converting enzyme inhibitors/angiotensin receptor blockers, and corticosteroids

## Discussion

In this large diverse sample of Hispanic/Latino adults, elevated serum aminotransferase levels (a biomarker of NAFLD) were associated with prevalent albuminuria, and this association was explained by metabolic syndrome. In contrast, we found no association between elevated aminotransferase levels and prevalent low eGFR. The study also showed that advanced liver fibrosis was associated with prevalent albuminuria independent of metabolic syndrome and other traditional risk factors.

NAFLD may be a feature of metabolic syndrome; however, it is not defined by the National Cholesterol Education Program-Adult Treatment Panel (NCEP-ATP) as a metabolic syndrome trait [27]. This perspective is supported by a study reporting that 90 % of NAFLD is a direct result of insulin resistance as a consequence of abdominal adiposity and diet [28]. This seems consistent with the findings from this study showing higher values of waist circumference and HOMA-IR among individuals with elevated aminotransferase levels than those without elevated aminotransferase levels. In the setting of insulin resistance, there is an increase in peripheral lipolysis, which leads to influx of free fatty acids into the liver [29]. In addition, insulin resistance induces intrahepatic triglyceride production [30]. The result is an increase in triglyceride content, which can progress to nonalcoholic steatohepatitis [31].

Consistent with our study, an analysis of NHANES data found an association between ultrasonography-diagnosed NAFLD and CKD after adjusting for age, sex, and race but not after adjusting for features of metabolic syndrome [10]. Similarly, a meta-analysis reported a null association between ultrasonography-diagnosed NAFLD and CKD after adjusting for metabolic syndrome in a European population [9]. Our results are also consistent with a recent study reporting no association between elastography-diagnosed NAFLD and CKD measures in the US population, but a significant association with advanced liver fibrosis [32]. Similar findings were also noted in the Chinese population, where albuminuria was significantly associated with advanced fibrosis, but not with steatosis [33]. The fact that the association between elevated aminotransferase levels and prevalent albuminuria was explained by metabolic syndrome in this study supports the hypothesis that both conditions share metabolic syndrome as a risk factor. As such, public health interventions that target metabolic syndrome in the Hispanic/Latino population would likely reduce the burden of both NAFLD and CKD. This perspective is reinforced by a post-hoc analysis of a randomized controlled trial showing an improvement in both NAFLD histology and kidney function following 1-year lifestyle modification [34].

This study found that the prevalence of elevated aminotransferase levels varied by Hispanic/Latino background with individuals of Mexican background accounting for nearly half of the prevalence. Previous studies have also reported that hepatic steatosis and NAFLD are more frequent among Mexican compared to all other racial/ethnic groups [2, 3]. While elevated aminotransferase levels may reflect NAFLD caused by obesity and insulin resistance, some elevated aminotransferase levels can also occur in genetic disease processes. In particular, prior research has demonstrated that patatin-like phospholipase domain containing 3 gene (PNPLA3 at Chr22q13.3) variants are associated with elevated serum aminotransferase levels [35–37] and heightened risk for NAFLD [26, 38]. It has been reported that PNPLA3 variants are more frequent among individuals with Mexican ancestry [39] and may contribute to the higher prevalence of elevated aminotransferase levels observed in Mexican population.

The strengths of this study include the representation of diverse Hispanic/Latino backgrounds and the systematic measurement of participant characteristics and co-morbidities. In addition, low GFR was defined using a cystatin C-based equation (i.e., not dependent on muscle mass) [40], and findings were consistent when we re-analyzed the data using creatinine-based GFR estimating equations. This study also has limitations. First, the aminotransferase thresholds used to define NAFLD may have led to misclassification. No aminotransferase thresholds exist for predicting NAFLD [41] and NAFLD may be present in the setting of normal serum aminotransferase levels [42], but higher serum aminotransferase levels generally indicate liver inflammation or damage. The thresholds used to define elevated serum aminotransferase levels have been utilized in previous studies [14, 43]. However, when we examined the association between elevated aminotransferase levels and CKD measures using different aminotransferase thresholds, we found similar results. Further, the patterns of the associations between aminotransferase levels and CKD measures persisted when we re-analyzed the data using serum aminotransferase as continuous variables. Second, laboratory testing for viral hepatitis was not performed for all HCHS/SOL participants, thus this study cannot be said to accurately exclude all cases of viral hepatitis. However, possible incomplete exclusion of individuals with viral hepatitis would not have biased the results of this study given that the prevalence of hepatitis B virus infection and hepatitis C virus in the United States is less than 1 and 2 %, respectively [1]. Third, the use of a cross-sectional design precluded us from assessing the temporal relationship between suspected NAFLD and CKD measures because it is possible that participants with elevated liver enzymes have a higher propensity for developing CKD.

## Conclusions

This study found that elevated serum aminotransferase levels were not independently associated with low GFR, but were significantly associated with higher odds of prevalent albuminuria after adjusting for demographic characteristics only. The later association was attenuated after adjusting for metabolic syndrome, thus suggesting that the two conditions may share metabolic syndrome as a mechanism.

## Supplementary Information

**Additional file 1:**

**Additional file 2:**

## Data Availability

The data analyzed during the current study are not publicly available due confidentiality but are available from the corresponding author on reasonable request.
