# Supplementary material for: Association of elevated serum aminotransferase levels with chronic kidney disease measures: hispanic community health study/study of latinos
Source: BMC Nephrol. 2021 Sep 7;22:302. doi: 10.1186/s12882-021-02483-y (PMC8422630; doi:10.1186/s12882-021-02483-y)
Supplement: Supplementary file 1 — Additional file 1: [file 12882_2021_2483_MOESM1_ESM.docx]

Supplemental Table 1: Odds Ratio and 95% confidence intervals of the association between elevated aminotransferase levels and low eGFR (eGFR calculated from CKD-EPI creatinine-cystatin C equation)

| Regression models | OR and 95%CI | P-value |
| --- | --- | --- |
| Unadjusted model | 0.74 (0.50, 1.12) | 0.155 |
| Model 1 | 1.05 (0.70, 1.58) | 0.809 |
| Model 2 | 0.92 (0.61, 1.40) | 0.702 |
| Model 3 | 0.97 (0.63, 1.49) | 0.882 |

- Elevated aminotransferase levels defined as aspartate aminotransferase (AST)> 37 U/L or alanine aminotransferase (ALT)> 40 U/L in men and AST or ALT > 31 U/L in women.
- Low eGFR defined as eGFR < 60 ml/min/1.73 m^2^.
- Model 1 adjusted for age, sex, Hispanic/Latino background, and study site.
- Model 2 adjusted for age, sex, Hispanic/Latino background, study site, and metabolic syndrome.
- Model 3 adjusted for age, sex, Hispanic/Latino background, study site, metabolic syndrome, education attainment, alcohol consumption, cigarette smoking, having health insurance, use of angiotensin converting enzyme inhibitors/angiotensin receptor blockers, and corticosteroids.
