# Supplementary material for: Association of elevated serum aminotransferase levels with chronic kidney disease measures: hispanic community health study/study of latinos
Source: BMC Nephrol. 2021 Sep 7;22:302. doi: 10.1186/s12882-021-02483-y (PMC8422630; doi:10.1186/s12882-021-02483-y)
Supplement: Supplementary file 2 — Additional file 2: [file 12882_2021_2483_MOESM2_ESM.docx]

Supplemental Table 2: Odds Ratio and 95% confidence intervals of the association between aminotransferases (as continuous variables) and low eGFR (eGFR calculated from CKD-EPI cystatin C equation) and increased urine albumin-to-creatinine ratio (UACR)

| Regression models | Low eGFR | | Increased UACR | |
| --- | --- | --- | --- | --- |
|  | Alanine aminotransferase (ALT) | Aspartate aminotransferase (AST) | Alanine aminotransferase (ALT) | Aspartate aminotransferase (AST) |
|  | OR and 95% | OR and 95% | OR and 95% | OR and 95% |
| Unadjusted model | 0.99 (0.98, 1.00) | 0.99 (0.98, 1.01) | 1.00 (1.00, 1.00) | 1.01 (1.00, 1.01) |
| Model 1 | 0.99 (0.97, 1.01) | 0.99 (0.97, 1.01) | 1.00 (1.00, 1.01) | 1.01 (1.00, 1.01) |
| Model 2 | 0.99 (0.98, 1.01) | 0.99 (0.97, 1.01) | 1.00 (0.99, 1.00) | 1.00 (1.00, 1.01) |
| Model 3 | 0.99 (0.97, 1.00) | 0.99 (0.97, 1.01) | 1.00 (0.99, 1.00) | 1.00 (1.00, 1.01) |

- Low eGFR defined as eGFR < 60 ml/min/1.73 m2.
- Increased UACR defined as UACR >17 mg/g in men and >25 mg/ g in women.
- Model 1 adjusted for age, sex, Hispanic/Latino background, and study site.
- Model 2 adjusted for age, sex, Hispanic/Latino background, study site, and metabolic syndrome.
- Model 3 adjusted for age, sex, Hispanic/Latino background, study site, metabolic syndrome, education attainment, alcohol consumption, cigarette smoking, having health insurance, use of angiotensin converting enzyme inhibitors/angiotensin receptor blockers, and corticosteroids.
